# Kidney Transplantation in Case of Renal Graft with Multiple Arteries: Challenges and Long-Term Results of Microsurgical Anastomosis Between Lower Polar Renal Artery and Inferior Epigastric Artery

**DOI:** 10.3390/medicina61091645

**Published:** 2025-09-11

**Authors:** Matteo Zanchetta, Gian Luigi Adani, Andrea Della Penna, Martina Guthoff, Vittorio Cherchi, Silvio Nadalin

**Affiliations:** 1 Unit of General Surgery and Surgical Oncology, Department of Medicine, Surgery and Neurosciences, University Hospital of Siena, 53100 Siena, Italy; matteozanchetta000@gmail.com; 2 Kidney Transplant Unit, Department of Medicine, Surgery and Neuroscience, University Hospital of Siena, 53100 Siena, Italy; 3 Department of General, Visceral and Transplant Surgery, University Hospital of Tübingen, 72016 Tübingen, Germany; 4 Department of Diabetology, Endocrinology, Nephrology, University Hospital of Tübingen, 72016 Tübingen, Germany; 5 General Surgery Clinic and Liver Transplant Center, University Hospital of Udine, 33100 Udine, Italy

**Keywords:** kidney transplant, vascular anastomosis, microsurgery, polar artery, epigastric artery, vascular reconstruction, transplant outcome, end-to-end anastomosis, living donor, deceased donor

## Abstract

*Background and Objectives:* In the current era of solid organ transplantation, the gap between available donors and patients on the waiting list is widening. Worldwide, surgeons are confronted with the challenge of optimizing the utilization of renal grafts, including the presence of multiple renal arteries (MRA), occurring in 20% to 30% of cases. The presence of a lower polar artery (LPA), which provides a significant vascular contribution to both the lower renal parenchyma and the upper urinary tract, constitutes an additional challenge, but its preservation is fundamental for the outcome of the kidney transplant (KT). The end-to-end (E/E) anastomosis with the recipient’s inferior epigastric artery (IEA) has been rarely reported in the literature, with variable results. The aim of this study is to report on technical aspects as well as on short- and long-term outcomes of this reconstruction in KT. *Materials and Methods:* A retrospective three-centre analysis was conducted on 13 KTs in which the graft’s LPA was anastomosed E/E with the recipient’s IEA. *Results:* Following an average follow-up period of 84 months, the patient and graft survival rate was 100%. Neither vascular nor urological complications were observed. *Conclusions:* In the event of KT with LPA, an E/E anastomosis with IEA performed with microsurgical technique is safe and provides excellent long-term results.

## 1. Introduction

**Figure 1:**
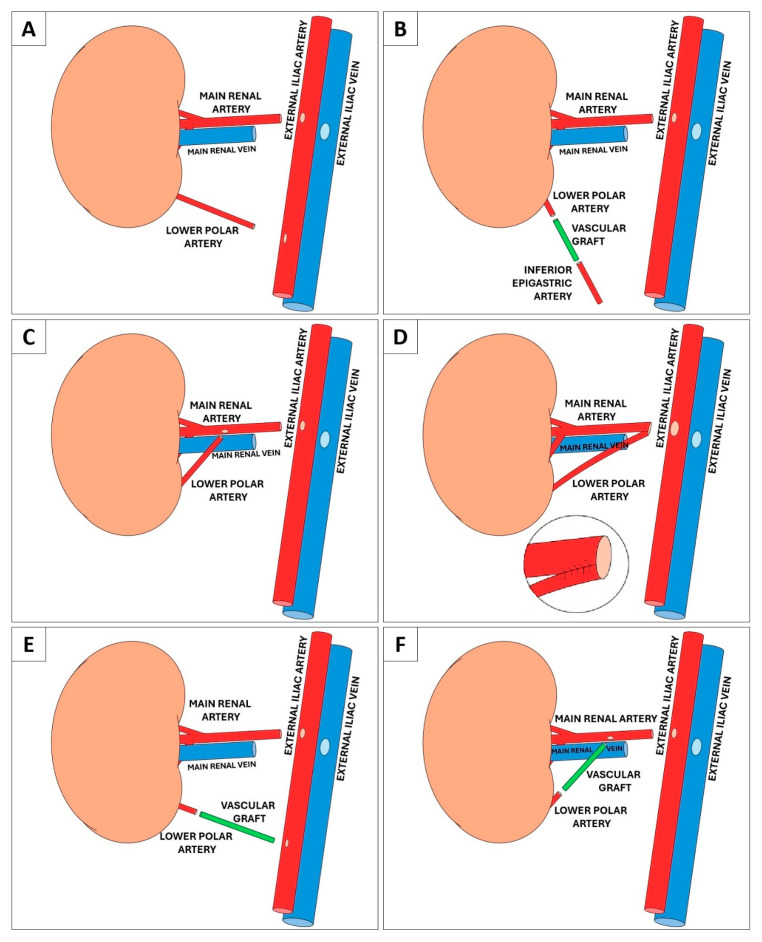
Techniques of reconstruction of the LPA during KT reported in the literature. (**A**) End-to-side anastomosis of the LPA on the external iliac artery [32]. (**B**) End-to-end anastomosis between the LPA and the IEA with an interposed vascular graft [35,36]. (**C**) End-to-side anastomosis of the LPA on the main renal artery [32]. (**D**) Suturing a single end ostium between the main renal artery and the LPA, anastomosed on the external iliac artery [32]. (**E**) End-to-side anastomosis of the LPA through an interposed vascular graft on the external iliac artery [30,36,37,38]. (**F**) End-to-side anastomosis of the LPA through an interposed vascular graft on the main renal artery [36,39].

For decades, kidney transplantation (KT) has been the first-line treatment for patients with end-stage renal disease (ESRD) [1,2]. However, with the increase in ESRD patients [3,4] and longer life expectancy, the gap between available donors and patients on the waiting list is widening [5], making efficient use of available grafts paramount. At a time when there is an ongoing shortage of organs available for transplantation, surgeons are faced with many challenges, including complex vascular variability of the graft, such as multiple renal arteries (MRA). The presence of MRA is the most common form of renal vascular anomaly, occurring in 20% to 30% of cases [6,7,8]. In the past, the presence of supernumerary arteries was considered a relative contraindication to both living (LD) and deceased donor (DD) KT. However, years of observation have shown that well-performed reconstructive and repair procedures can lead to successful transplantation of an organ with various forms of anomalies, and today most studies agree that MRA should no longer be considered a problem [9,10,11,12,13,14]. Polar arteries are anatomical variants of renal vasculature that are uncommon but well described in the literature [15,16]. It has been suggested that the size of the accessory artery could be used to decide whether or not to ligate it during KT [17]. The upper polar arteries (UPA) usually supply small amounts of renal parenchyma [18]. Ligation of the UPAs, when their calibre is deemed not worthy of reconstruction, has been shown to be safe [19]. On the other hand, the lower polar arteries (LPAs), despite their lesser calibre, frequently provide a more than valuable vascular contribution to both the lower renal parenchyma and the upper urinary tract [20], as shown in a recent study [21]. Therefore, reconstruction of the LPA in KT is essential to reduce the risk of ureteral necrosis and ensure a satisfactory perfusion of the graft parenchyma [20,22]. Various reconstruction techniques have been described in the literature (Figure 1A–F) (Table 1), among which there is an E/E anastomosis between the donor’s LPA and the recipient’s inferior epigastric artery (IEA).

The objective of this study is to present a report on one of the most extensive series of LPA-IEA reconstructions in MRA-KT, focusing on the technical aspects of the reconstructions, as well as on the short- and long-term outcomes.

**Table 1:** Review of the literature of papers reporting the LPA-IEA anastomosis in KT (in chronological order, including our own experience). Under Cases, the number of LPA-IEA considered in the study, and in brackets, the overall number of MRA-KTs described in the publication. Under FU, the study average FU or the range of FU in months. E/E: end-to-end. IEA: inferior epigastric artery. KT: kidney transplant. LPA: lower polar artery. MRA: multiple renal arteries.

Author	Year	Cases	FU	Conclusions
Chautems et al. [23]	2000	3	0.75, 16, 216	The use of the IEA may be an appropriate alternative in the revascularization of a lower-pole renal artery.
Kumar et al. [24]	2001	10 (51)	25.1	Kidney grafts with MRAs can be used with excellent results.
Wolters et al. [22]	2001	3	26	IEA seems to be a useful alternative in KT.
Makiyama et al. [25]	2003	3 (96)	12	Allografts with MRAs can be used successfully in KT in the short term.
Başaran et al. [26]	2004	28 (79)	60	Kidney grafts with MRAs can be used with excellent results.
El-Sherbiny et al. [27]	2007	15 (48)	28.8	The use of the IEA was as effective as intracorporeal and extracorporeal bench surgery for the anastomosis of MRAs, without an increase in the incidence of relevant complications.
Gomes et al. [28]	2009	5 (7)	12–60	The IEA anastomosis to a small polar vessel in an MRA kidney can be successfully used in KT with good outcomes.
Amirzargar et al. [29]	2013	68	68.4	Kidneys with MRAs can be safely and successfully utilized for KT by anastomosing the accessory arteries to the epigastric arteries.
Antonopoulos et al. [30]	2014	21	43.8	Allografts with MRAs can be used successfully in a living-related renal transplantation program. Bench reconstruction should be performed whenever possible. For reconstruction of an accessory vessel, the IEA with sequential revascularization is recommended.
Lim et al. [31]	2016	7 (12)	12	Living donor kidneys with MRA can be transplanted with favourable outcomes.
Yamanaga et al. [32]	2018	21 (311)	62	Alternative techniques to the E/S with the main renal artery anastomosis should be used for accessory arteries that require revascularization.
Kumar et al. [33]	2022	6 (19)	1	Renal transplantation is not inferior in multiple-vessel allograft patients.
Ostrowski et al. [34]	2022	1	Until discharge	Well-carried out reconstructive and repair procedures can result in a successful transplant of an organ with various forms of anomalies.
Actual experience	2025	13	84	Neither vascular nor urological complications. Both patient and graft survival rates are 100%. In MRA KT with an LPA, an E/E anastomosis with IEA performed with microsurgical technique is safe and provides excellent long-term results.

## 2. Materials and Methods

**Figure 2:**
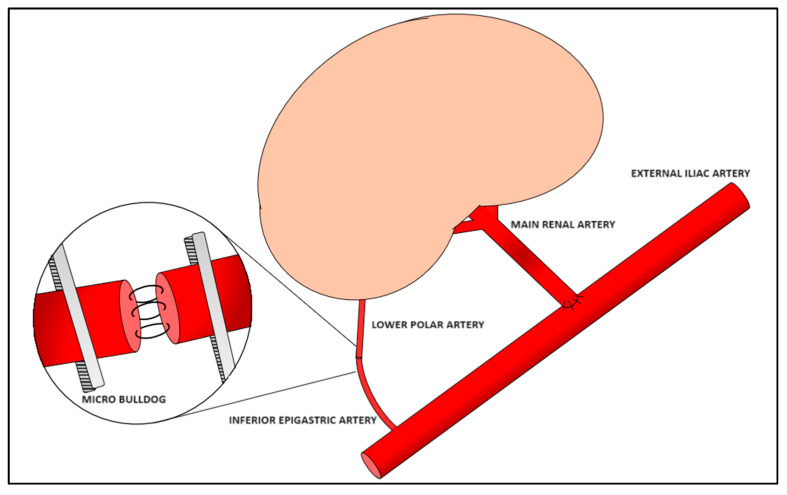
Schematic representation of the LPA-IEA end-to-end anastomosis.

**Figure 3:**
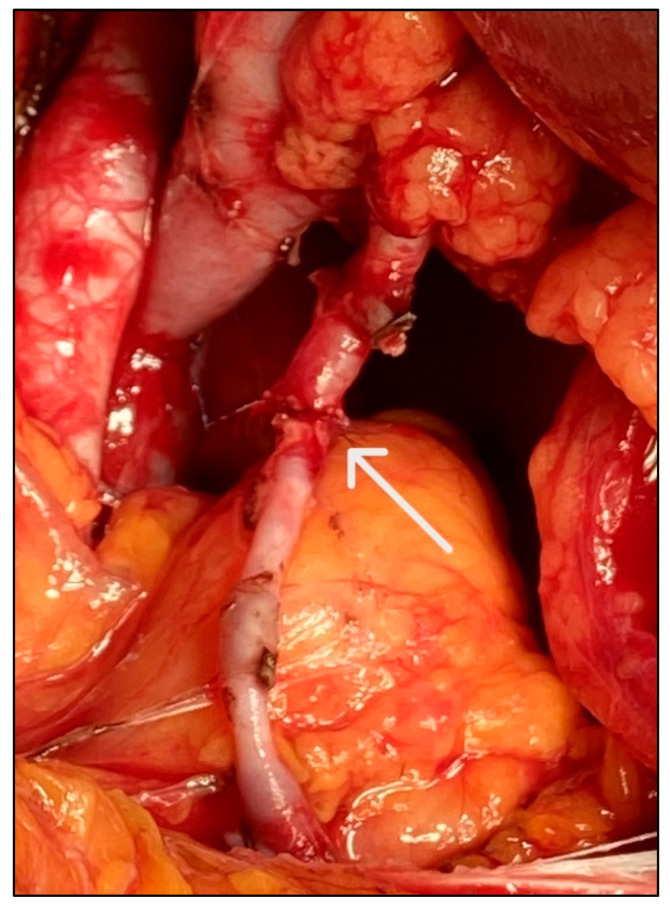
Intraoperative image: LPA-IEA anastomosis (arrow).

We performed a retrospective analysis of 13 KTs in which the graft LPA was anastomosed end-to-end to the IEA of the recipient, performed from 2008 to 2022 at the Department of Transplant Surgery of the University Hospital Tübingen in Germany (n = 9), at the Department of Kidney Transplant Surgery of Azienda ospedaliero-universitaria Senese in Italy (n = 2), and at the General Surgery Clinic and Transplant Centre, University-Hospital of Udine in Italy (n = 2) (Figure 2). A Gibson incision and deeper tissue dissection were employed to gain access to the iliac fossa, thus exposing the external iliac vessels; a median laparotomic incision was used for the patient who underwent a simultaneous nephrectomy. The external iliac artery and vein were then partially freed from surrounding tissues. Subsequently, the recipient’s IEA was exposed laterally to the posterior rectus abdominis sheath and moved down towards its origin from the external iliac artery. It is crucial to ensure an adequate length of IEA exposure and ligate minor side branches to guarantee the safety and efficacy of the anastomosis to the LPA. The length of the LPA may vary according to the organ recovery procedure, and it is therefore essential to avoid potentially dangerous tension or kinking of the anastomosis. Once the optimal length for vascular reconstruction has been determined, the IEA was excised and its cranial portion ligated, while the caudal stump was clamped with a micro-Bulldog clamp. The LPA was also clamped with a micro-Bulldog clamp to prevent bleeding after reperfusion of the main vessels. The graft’s main vein and main artery were then end-to-side anastomosed to the recipient’s external iliac vein and artery, respectively. Primary reperfusion was then achieved by declamping the main vessels, thus reducing the general ischemic time of the graft. The LPA and IEA were then briefly declamped and flushed. Subsequently, the single stitches end-to-end LPA-IEA anastomosis was performed with 6-0 (n = 4), 7-0 (n = 8), or 8-0 (n = 1) Prolene sutures, using 5× magnifying loupes (Figure 3). Finally, the Bulldog clamps were removed, according to the sequential reperfusion technique, allowing the secondary reperfusion to occur. Doppler US was performed intraoperatively, during hospitalization, before discharge, and at follow-up (FU), with routine laboratory blood tests, including markers of renal function (e.g., creatinine). All patients received 100 mg/day of Acetylsalicylic acid from the second postoperative day. After discharge, FUs took place at approximately one month, three months, and six months postoperatively, and then yearly. Our analysis focused on long-term patient and graft survival and the rate of postoperative complications in MRA KTs performed with this specific microsurgical vascular technique. The complications were classified according to the Dindo–Clavien classification (CDC) of surgical complications [40]. We considered vascular complications (e.g., arterial thrombosis, anastomotic hemorrhage, etc.), urologic complications (e.g., necrosis of the ureter, urine leak, etc.), and other complications (e.g., postoperative infections, lymphocele, acute or chronic rejection, delayed graft function [DGF], primary non-function (PNF), graft loss, etc.). Delayed graft function was defined as the need for one or more dialysis sessions in the first week after transplantation. Primary non-function was defined as a failure in the initial stages after transplantation, resulting in a permanent lack of graft function despite the presence of adequate perfusion (confirmed by US examination), therefore necessitating continuation of dialysis and/or the explantation of the graft. Graft loss was defined as cessation of function (i.e., need for permanent dialysis).

## 3. Results

Seven grafts were obtained from living donors, and six from deceased brain-dead (DBD) donors. The average age of donors was 55.1 years (n = 10, range 49–67). All grafts had one main RA, one smaller LPA, and one renal vein. Among recipients, six were males and seven were females, with average age and BMI, at the time of surgery, of 51.75 years (range 21–72) and 27.1 kg/m^2^ (range 20.8–31.3), respectively (the pediatric patient was excluded from the calculation of average age and BMI) [Table 2]. Prior to KT, seven patients were undergoing haemodialysis, one peritoneal dialysis, and the remaining five were pre-emptive KTs. One patient had previously received a KT, and one patient underwent a simultaneous native kidney nephrectomy. Indications for KT are reported in Table 3. All KTs were AB0 compatible (A = 6, B = 3, AB = 2, 0 = 2). The average cold ischemia time was 119.4 (range 58–157) minutes in the case of LD-KT, and 1102.3 (range 680–1749) minutes in DD-KT. The average warm ischemia time was 31.5 (range 21–40) minutes, and the average additional warm ischemia time between main artery declamping and reperfusion of LPA-IEA was 5 (range 4–7) minutes. Doppler US performed intraoperatively, during hospitalization, and at discharge was always unremarkable. At discharge, the average values of serum creatinine (n = 12) and BUN (n = 8) were 1.3 (range 0.7–2.13) mg/dL and 46.75 (range 30–64) mg/dL, respectively.

No vascular nor urological complications occurred throughout the entire FU period. Other complications were as follows [Table 4]: acute graft rejection (n = 1), successfully treated with steroids; lymphocele (n = 1), requiring temporary ureteral stenting and surgical evacuation; abdominal parietal bleeding (n = 1), requiring surgical revision; bleeding of unknown origin (n = 1), spontaneously resolved without surgical revision; urinary tract infection (n = 2), successfully treated with antibiotics; DGF (n = 1) resolved within a session of dialysis. Regarding the FU, a 13-year-old patient was excluded from further calculations for the study due to her death three months after the KT, caused by the unexpected early recurrence of the primary haematological disease, even though the graft was still functioning. Average FU (n = 12) was 83.75 months (range 18–155 months) [Table 5]. At the last FU, average serum creatinine (n = 12) and BUN (n = 8) values were 1.5 mg/dL (range 0.7–2.43) and 65.1 mg/dL (range 30–86), respectively. At the last available FU (n = 12), patient and graft survivals were both 100%.

## 4. Discussion

Considering the ever-increasing requirement for donated grafts and the consequent necessity to maximize their survival chances, surgeons are faced with many challenges, including complex vascular variants of the graft, such as MRA. The presence of MRA is the most common form of renal vascular anomaly, occurring in between 20% and 30% of cases [6,7,8]. In these cases, the surgeon must use special vascular surgery techniques to perform the KT, including microsurgery, to anastomose small arteries, but revascularization of these small-caliber vessels can be challenging. Accessory arteries supplying the upper renal pole, according to their size, could be ligated, avoiding the unnecessary risk of complications associated with multiple anastomoses at the expense of a small amount of nephron mass [19]. On the other hand, to prevent ureteral necrosis, segmental renal infarction, postoperative hypertension, graft rupture, and calyceal fistula formation, it is important to preserve accessory arteries that supply the lower renal pole or significant areas of renal parenchyma [22,41].

The choice of reconstruction technique is influenced by the diameter and length of the LPA, the recipient’s vascular anatomy and status (e.g., presence of atherosclerosis and calcifications rendering the IEA unsuitable for use), and the surgeon’s expertise (see also Figure 1). This accessory artery can be anastomosed end-to-side with the main renal artery either along its course or to one of its hilar branches [42]. However, some authors strongly advise against this anastomosis due to the risk of manipulating the main renal artery during surgery and compromising its functionality by altering its anatomy [30]. Furthermore, it has been reported that the end-to-side anastomosis between the main RA and the LPA results in worse outcomes than other vascular reconstruction techniques for LPA in KT [32]. If the length and size of the LPA are sufficient, it can be joined to the main renal artery in a single terminal lumen, or it may be anastomosed to the aortic patch [42]. Alternatively, the LPA may be anastomosed end-to-side along the iliac axis [32]. When the LPA is too far from the main RA or severe atherosclerosis affects the greater vessels, an end-to-end anastomosis with the recipient’s IEA can be a viable alternative [22,30,34]. As a further alternative, an interposed vascular graft can be used to connect the LPA to the main RA, the iliac axis, or the IEA [35,38] when the accessory vessel is too short.

Reconstruction with an end-to-end LPA-IEA anastomosis has been seldom described with accuracy in the literature and has been associated with a variable percentage of vascular or urological complications, such as LPA thrombosis, anastomotic bleeding, partial infarction, postoperative hypertension, urinary fistula, ureteral necrosis requiring intervention, and graft loss [22,27,29,30]. This vascular reconstruction technique has been described as “supercharging vascular augmentation”, i.e., using a different, distant vascular source to supply a specific tissue [43].

Multiple vascular anastomoses require a prolonged ischemic time, which may increase the incidence of acute tubular necrosis (ATN) according to some authors [44]. However, other authors disagree, suggesting that in MRA KT the incidence of postoperative ATN or surgical complications does not exhibit any differences [45,46].

The anastomosis of the LPA to the IEA offers several advantages. Firstly, the graft’s function is minimally affected because the anastomosis may be performed after declamping the main vessels. Secondly, the main RA is exposed to the minimum necessary manipulation required only for its own anastomosis to the iliac axis. Finally, the end-to-end anastomosis facilitates a more natural blood flow in the LPA. We performed the LPA-IEA anastomosis after declamping the main vessels, according to the sequential reperfusion technique [24], to reduce the warm ischaemic time and thus the potential complications of a complex implantation [44,47,48]. By sequentially anastomosing the LPA to the IEA, we protected the main RA from potential complications and reduced the total graft warm ischemia time. Although some may disagree with their use in principle [49], we believe that MRA grafts with LPA can be successfully transplanted, as long as such a critical accessory vessel is properly reconstructed by an expert surgeon. The use of surgical loupes has been found to be fundamental in performing these microsurgical anastomoses. The long-term survival of single-artery KTs, which are not subjected to the higher risk of complications resulting from the multiple anastomoses, is comparable to the data presented here [39,50]. Despite the significant risk of a higher incidence of postoperative vascular and urological complications with single-artery versus MRA KT, as reported by a large meta-analysis, our patients did not experience any such complications [51].

Based on the positive outcomes of our multicenter experience, with no major vascular or urological complications over a long FU period, we conclude that in renal allografting, safe reconstruction of the LPA can be performed with a microsurgical end-to-end anastomosis to the IEA, carrying a negligible chance of postoperative complications. It is important to note that this type of anastomosis is seldom performed. The strength of this study lies in the duration of the FU, which, to the best of our knowledge, is the longest average period reported to date.

## 5. Conclusions

At a time when there is an ever-increasing shortage of organs available for transplantation, surgeons are faced with many challenges, including the complex vascular variability of the graft, such as MRA. While the UPA can often be sacrificed without consequences, the LPA plays a fundamental role in the outcome of KT. There are several reconstruction techniques for LPA in KT. Among them, the LPA-IEA anastomosis is a rare one, and the literature reporting its long-term outcome is scarce. The anastomosis of the LPA to the IEA provides several advantages. As shown in our study presenting a long-term FU, this anastomosis, when accurately performed by a surgeon with microsurgery expertise, carries excellent and durable results, simultaneously protecting the main RA from potential complications and reducing the whole graft ischemic time in MRA KTs.

## Figures and Tables

**Table 2:** Recipients’ data. Age and BMI were calculated for the twelve adult patients. The FU (months) was re-calculated from twelve patients, excluding the paediatric patient who had died because of a recurrence of the primary haematologic disease three months after KT.

Patient	Age	Sex	BMI	Blood	Donor	Fu
1	72	1	28.7	A	Living	155
2	59	0	31.3	A	Living	148
3	27	0	21.8	0	Living	147
4	45	0	20.8	B	DBD	146
5	55	0	31.2	0	Living	67
6	13	0	-	AB	Living	3
7	47	0	27	A	DBD	24
8	21	1	22.3	A	Living	64
9	39	1	28.9	AB	Living	18
10	65	1	27.1	A	DBD	60
11	67	1	28	B	DBD	60
12	70	0	27.8	B	DBD	60
13	54	1	31	A	DBD	60
Average	51.75		27.15			84

**Table 3:** Indication for kidney transplantation.

Number	Main Pathology Leading to KT
4	Glomerulonephritis
3	Autosomal dominant polycystic kidney disease
1	Pre-emptive for renal failure in Ewing’s sarcoma
1	Mesangioproliferative IgA nephritis
1	Renal failure after therapy for acute myeloid leukemia
1	Hereditary nephropathy
1	Alport syndrome
1	Chronic tubulointerstitial nephritis and benign nephrosclerosis

**Table 4:** Postoperative complications classified according to the CDC.

Postoperative Complication	Number	CDC Grade
Delayed graft function resolved within one session of dialysis	1	IV a
Abdominal parietal bleeding requiring surgical revision	1	III b
Lymphocele requiring temporary ureteral stenting and surgical evacuation	1	III a
Acute graft rejection treated with steroids	1	II
Abdominal bleeding of unknown origin spontaneously resolved	1	II
Urinary tract infection treated with antibiotics	2	II

**Table 5:** Clinical data regarding the KTs and renal function. CIT: cold ischemia time (minutes); WIT: warm ischemia time (minutes); creatinine measured in mg/dL; BUN: blood urea nitrogen (mg/dL).

Patient	CIT	WIT	Additional WIT for LPA-IEA	Creatinine at Discharge	BUN at Discharge	Most Recent Creatinine	Most Recent BUN
1	58	38	4	-	-	1.7	77
2	75	38	5	1.1	46	0.9	30
3	132	21	4	0.8	30	0.7	28
4	870	24	6	1.5	41	1.3	65
5	120	31	6	1.2	61	1.8	79
6	157	33	7	0.7	54	-	-
7	680	35	6	1.5	43	1.1	53
8	144	24	6	1.1	35	1.8	86
9	150	22	5	1.8	64	2.2	38
10	1096	40	4	2.13	-	1.7	-
11	1485	32	5	1.09	-	0.97	-
12	734	37	4	1.27	-	1.45	-
13	1749	35	5	1.45	-	2.43	-
Average			5.13	1.3	46.75	1.5	57

## Data Availability

Patients’ data registry of Universitätsklinikum Tübingen, Tübingen, Germany.
